# Teaching stress reduction techniques including biofeedback for managing stress in medical students

**DOI:** 10.1192/j.eurpsy.2024.1406

**Published:** 2024-08-27

**Authors:** R. Krishnan, K. Kósa

**Affiliations:** ^1^Department of Behavioural Sciences, University of Debrecen, Debrecen, Hungary; ^2^Greenwich Mental Health Liaison Team, Oxleas NHS Foundation Trust, London, United Kingdom

## Abstract

**Introduction:**

Medical students have been under immense pressure throughout their studies, impacting their mental health and academic performance. Stress reduction is a fundamental skill that all students require to manage their studies and lives efficiently. Biofeedback devices providing information about physiological states have been shown to aid stress reduction. Methods to reduce stress should be taught to medical students to help them tackle the challenges of medical school.

**Objectives:**

Our goal was to teach stress reduction methods such as extracurricular activities and paced breathing aided by biofeedback training and its application in simulated healthcare situations to medical students.

**Methods:**

15 medical students who completed medical physiology were recruited for an elective course of 7 sessions on practical techniques in stress management. One credit was offered to those who completed the course requirements consisting of participation in sessions and individual biofeedback training.

Sessions (classes) consisted of presentations on good sleeping and eating patterns, group simulations of stressful hospital environments, visiting a science centre with interactive displays, an orchestra performance, and nature walks. Before biofeedback training, heart and respiration rates were taken individually by a biofeedback device during the first week of the course. Data was processed using a code created in statistical software. Heartbeats per minute and heart rate variability (HRV) for every 10 seconds were calculated and plotted on a graph. Two measurements were taken with each student: a baseline measurement for 10 minutes and another measurement during controlled breathing paced at 6 breaths per minute for 15 minutes, of which the first 10 minutes were used for calculation and plotting. Students provided narrative feedback in an essay submitted after the course was completed.

**Results:**

5 males and 10 females from years 2-5 registered for the elective, and 12 participated in individually scheduled sessions. Heart beats per minute decreased, whereas HRV increased during paced breathing sessions in 83% of them. Most students reported feeling calm and drowsy during the sessions, and 2 students fell asleep by the end.

Feedback from 11 students showed that the music session and the science centre visit were the highlights throughout the elective. Improvements recommended were to have a consistent time slot for all sessions and fewer simulations.

**Conclusions:**

In concert with the literature, biofeedback training seems to be a feasible and effective method for relaxation in medical students. This method could be offered as part of mental health services for students. Data could be used to follow students’ progress and identify those requiring extra support. Providing them with avenues to de-stress while emphasizing activities outside medicine could boost their confidence and improve their coping skills.

**Disclosure of Interest:**

None Declared

